# Family Planning practices among the parents of beta thalassemia major patients in Makran division, Balochistan: A cross-sectional study

**DOI:** 10.12669/pjms.41.1.10263

**Published:** 2025-01

**Authors:** Uzair, Mehtab Malik, Zarnum Gul, Jeehand Bohair, Syeda Khalida Naeem, Noman Sadiq

**Affiliations:** 1 Uzair, Final Year MBBS Student, Makran Medical College, Turbat, Pakistan; 2 Mehtab Malik, Final Year MBBS Student, Makran Medical College, Turbat, Pakistan; 3 Zarnum Gul, Final Year MBBS Student, Makran Medical College, Turbat, Pakistan; 4 Jeehand Bohair, Final Year MBBS Student, Makran Medical College, Turbat, Pakistan; 5 Syeda Khalida Naeem, MBBS, FCPS, MRCOG, Associate professor, Department of Obstetrics & Gynecology, Makran Medical College, Turbat, Pakistan; 6 Noman Sadiq, MBBS, M.Phil, Associate professor, Department of Physiology, Makran Medical College, Turbat, Pakistan

**Keywords:** Beta-thalassemia, Contraception, Family planning, Pakistan

## Abstract

**Objectives:**

To determine the prevalence of family planning practice among the parents of children affected with beta thalassemia major (BTM) and to determine the relationship between various factors and family planning practice in Makran division Balochistan.

**Methods:**

A cross-sectional observational study was conducted on 190 parents having BTM children registered in thalassemia care centers throughout the Makran division from May 2023 to October 2023. The structured questionnaire was used and data was collected using the non-probability convenience technique. SPSS 20 was used for analysis.

**Results:**

A total of 190 parents were interviewed and only 111 (58.42%) couples were practicing family planning. A significant relation was found between Family planning and monthly income, religious belief, parents with more than three children, and different districts of the Makran division. (p<0.05) The most common methods of contraception used by participating couples were oral contraceptive pills (OCPs) (18.4%) followed by barriers (16.8%). The predominant reason for not practicing family planning was the desire to have more children and religious beliefs.

**Conclusion:**

The religious beliefs, economic status of the family, area of residence, and total number of children play a significant role in family planning practices among the parents of children affected with BTM.

## INTRODUCTION

The word thalassemia is composed of thalas-, which means “sea”, and -emia means “blood”.^1^ This disorder is related to the population of the countries around the Mediterranean Sea and Indian subcontinent but now extends to other areas.^2^ Thalassemia major is the most common inherited blood disorder affecting 1.5% of the world’s population.^3^,^4^ According to rough estimation there are 2000 children with thalassemia major in Balochistan, Pakistan.^3^ Approximately 5 to 6 million children in Pakistan are suffering from thalassemia.^1^ About 3750000 carriers and 20000 patients of beta thalassemia major in Iran.^5^ The burden of thalassemia is high in India with 12000 newborns affected with BTM and 30 to 40 million carriers.^4^

Thalassemia is a blood disorder that affects the formation of the beta chain of hemoglobin that yields immature RBCs in the body which causes severe anemia and other blood disorders. The symptoms are managed by repeated blood transfusion and iron-chelating agents.^1^ Iron overload may cause failure of the heart that leads to death in BTM.^6^ To decrease the prevalence of thalassemia, prevention is the best method and for that different policies were applied in many states.^2^ Premarital screening program has an important role in dictating the prevalence of hereditary disorders and it is required to control the birth of a thalassemia child by preventing marriages among carriers.^7^,^8^

World Health Organization (WHO) defines family planning (FP) as, “the ability of individuals and couples to anticipate and attain their desired number of children and the spacing and timing of their births. It is achieved through the use of contraceptive methods and the treatment of involuntary infertility”.^9^ the main preventive strategy for thalassemia is proper family planning in couples with minor thalassemia. other preventions are genetic counseling before marriage and prenatal screening.^4^ Only 33.8% of married females had access to adopt family planning methods in Quetta.^9^ The contraceptive prevalence rate of Balochistan is 13.8% while in Sindh 25%, in Punjab 38.9% and 46% in Khyber Pakhtunkhwa.^10^ The current contraceptive prevalence rate of Pakistan is 30-35% while the president has promised to increase the contraceptive prevalence rate (CPR) to 50% by 2025.^11^ Among the 1.9 billion women of reproductive age (15-49 years), 842 million use contraceptive methods and 270 million have an unmet need for contraception.^9^

Despite of increasing incidence and lack of proper management, very limited data is available about thalassemia and its prevention in Pakistan. With that knowledge in mind, the current study was conducted to assess the family planning practices among parents with children affected with B thalassemia major and its related factors that restrict parents from adopting family planning methods in Makran Balochistan. Some states like Turkey and Cyprus have adopted the policy of stopping the birth of new thalassemia children but the government of Iran has made blood tests for pre-marriage and genetic counseling mandatory,^1^ So this study can be a guideline for improvising the current situation of having increased prevalence in Makran by not only identifying the indicator of highest prevalence but also would rectifying them by its appropriate implications and application of standard birth control practices.

## METHODS

A cross-sectional study was conducted in three cities of Makran division Balochistan; i.e. Turbat, Gwadar, and Panjgur from May 2023 to October 2023. The study was conducted on the parents of thalassemia major patients registered in Kech Thalassemia Care Center (KTCC) Turbat, Thalassemia Care Center Gwadar, and Panjgur. There are 540 registered families in these centers. The sample size of 225 was calculated using openEpi with 95% confidence level. Nonprobability convenience purposive sampling was used to collect the parents.

### Inclusion and Exclusion Criteria:

Parents of thalassemia-affected children visiting thalassemia centers on scheduled days were included in the study. Parents not willing to consent, parents of critically ill patients, separated or divorced partners, or single parents were excluded from the study. The structured questionnaire was used to collect the data and written informed consent was taken from all the study participants.

The questionnaire consisted of two sections, sociodemographic characteristics including name, age, address, ethnicity, religion, educational level, caste, and monthly income, total number of children, number of children suffering from thalassemia, the other section had questions about family planning practices and associated factors.

### Ethical Approval:

It was obtained from the ethical committee of the Makran Medical College Turbat under ref no. MMC/ERC/2023/May/01. The study was conducted under the supervision of the Department of Physiology and the Department of Obstetrics and Gynecology. The statistical analysis was performed on SPSS version 20.

## RESULTS

The data from 190 families was included in the study. Of 190 couple participants, 111 (58.42%) were practicing family planning. The mean age of the husband was 38.18± 9.78 and the mean age of the wife was 32.28±7.80. One hundred participants of the study belonged to Turbat, 59 from Gwadar, and 31 from Panjgur. In this study, 105 (55.3%) participants were residing in rural areas. The majority of mothers 120(63.2%) were illiterate while the literacy rate among fathers was 119(62.6%). One hundred seventeen (61.6%) parents were married to their 1^st^ cousin, 41 (21.6%) to 2^nd^ cousin and only 32 (16.8%) couples didn’t have any prior blood relation. The average number of children of these parents was 3.78±1.88, and among these thalassemia-affected children were 1.30±0.55.

The use of family planning methods in Panjgur, Gwadar, and Turbat was 74%, 66.10%, and 49% respectively. Thus the rate of family planning among the cities was significant (p=0.016). The monthly income of 44(23.2%) families was in the range of 5000-10,000, 56(29.5%) had a family income of 10,000 – 20,000 and 90(47.4%) earned above 20,000 which indicates that family planning decision significantly correlates with family income (p= 0.034). The parents with more than three children were more prone to use family planning (p=0.01). Some couples (37.9%) reported that family planning is a sin while 118(62.1%) do not consider it a sin. This Religious belief of parents significantly correlates with the use of contraception (p= 0.036) (Table-I).

**Table-I:** Parameters associated with family planning practices (N=190).

Parameter	Family planning practice		x^2^	p-value	Odd ratios with 95% Confidence interval

	Yes	No			
***Residence***					
Rural	56(295%)	49(258%)	2.501	0.1	0.89-2.88
Urban	55(289%)	30(15.8%)			
District					
Turbat	49 (25.8%)	51 (26.8%)	8.26	0.016*	
Gwadar	39 (20.5%)	20 (10.5%)			
Panjgur	23 (12.1%)	8 (4.2%)			
***Husband literacy***					
Literate	36 (18.9%)	35 (18.4%)	2.78	0.1	0.33-1.09
Illiterate	75 (39.5%)	44 (23.2%)			
***Wife literacy***					
Literate	66 (35.3%)	53 (27.9%)	0.898	0.3	0.40-1.36
Illiterate	44 (23.2%)	26 (13.7%)			
***Total number of child***					
1	7 (3.7%)	12 (6.3%)	9.133	0.01*	
2 to 3	39 (20.5%)	37 (19.5%)			
>3	65 (34.2%)	30 (15.8%)			
***Number of thalassemic children***					
1	82 (43.2%)	61 (32.1%)	0.894	0.6
2 to 3	28 (14.7%)	18 (9.5%)		
>3	1 (0.5%)	0 (0.0%)		
***Monthly income***					
5000-10000	19 (10.0%)	25 (13.2%)	6.763	0.034*	
10000-20000	32 (16.8%)	24 (12.6%)			
>20000	60 (31.6%)	30 (15.8%)			
***Religious beliefs***					
Sin	36 (18.9%)	36 (19.5%)	3.384	0.1	
Not a sin	75 (39.5%)	43 (22.1%)			
***Relation between couples***					
1^st^ cousin	65 (34.2%)	52 (27.4%)	1.34	0.5	
2^nd^ cousin	27 (14.2%)	14 (7.4%)			
Out of family	19 (10.0%)	13 (6.8%)			

* Chi-square (p<0.05)

Out of the total study population, 95.8% heard about family planning and 89.5% were aware of different methods of contraception. The most common methods of contraception used by participants are barriers (16.8 %) and OCPs (18.4%) followed by injectable (13.2%) (Table-II). The most prevalent reason for not using any family planning method was the desire to have more children while religious beliefs of considering it a sin were 2^nd^ major reason (Table-III).

**Table-II:** Contraceptive methods used by study participants (n=111).

Methods	frequency
OCPs	35 (18.4%)
Barriers	32 (16.8%)
Injectable	25 (13.2%)
Withdrawal	6 (3.2%)
Natural and behavior	5 (2.6%)
Jadelle/Implanon	3 (1.6%)
Tubal ligation	3 (1.6%)
Intrauterine device (IUD)	1 (0.5%)
Vasectomy	1 (0.5%)

**Table-III:** Reasons of not using contraception (n=79).

Reason	frequency
Desire of more children	32 (40%)
Religious issue	19 (24%)
Lack of knowledge	14 (17.7%)
Afraid of side effects	5 (6.3%)
Others	17 (21.5%)

## DISCUSSION

Beta Thalassemia major is an inherited blood disease due to a defect in the synthesis of the beta chain of hemoglobin leading to severe anemia in the affected person. There is no definitive cure for thalassemia but proper family planning in thalassemia minors is the most convenient and affordable method to prevent the birth of a thalassemia child. We have explored the relationship of various factors with family planning practice among the parents of children affected by BTM and a significant relationship was found between The religious beliefs, economic status of the family, area of residence, and total number of children.

More than half of the study participants were currently practicing family planning which is quite better in comparison to a study in India where only 44.8% of couples were using contraception.^6^ but insufficient to a study in Iran where the rate of family planning in affected couples was 96.0%^2^ similar results (51.8%) were reported in an Indian study.^12^ Our result showed that the rate of family planning varies in different districts of the Makran division. Family planning practices were relatively higher in Panjgur than in Gwadar and Turbat. This discrepancy may be seen due to the influence of different social and religious beliefs or the accessibility of family planning services in these cities.

Our study revealed that family planning is better in families who have a monthly income of more than 20000 with (p = 0.034) this is similar to the previous study which described that families with low economic status had a higher number of thalassemia-affected children (p= 0.035)^2^. Another study described the family planning trends and programming in Pakistan and stated that modern family planning depends on economic status, education, urban residency, and age.^13^ Use of contraceptives was significantly high (p=0.01) in parents with more than three children. The findings of our study are similar to the study conducted in Pakistan showed that contraceptive prevalence increased with the increasing number of children.^14^ In our study, some respondents stated that family planning is a sin. But despite this belief, half of these couples were currently practicing family planning, a study explored the factors affecting contraceptive use in Kenya and found that some participants believed that family planning is admissible under certain conditions as they advert it is allowed if it is necessary for the health of women and child.^15^

In this community majority of couples were cousins, as a previous study reported that 80% of couples with thalassemia-affected children are married to their first cousins, 7% are blood relatives, 6% are from the same caste, and only 4% are married out of the family. The risk of having genetic disease increases by 20 folds in first-cousin marriages therefore it is restricted in many countries.^16^ In contrast, only 18(15%) thalassemia children had evidence of cousin marriage of their parents and 85% were married to outsiders.^17^ In district Bannu parents of thalassemia, major patients were screened, and discovered that 74% of them were cousins and 26% were not relatives.^18^

The most common method was condoms (85%) followed by rhythm (81.5%) and withdrawal (80%) which is reported by a study that is conducted in Hyderabad Pakistan.^19^ In our study, we have seen that the most common methods of contraception by participants are OCPs followed by barriers and injectable. In our study, the most common reason for not using any form of contraception was the desire to have more children. while husband disapproval (74%) was the most frequent reason.^20^ Infrequent sex and fear of illness were the most common reasons for not using contraceptives in low and middle-income countries, but the frequency of each reason changed across various countries and different subgroups of women.^21^

### Limitation:

Limited time of visit to thalassemia centers. Other possible factors influencing family planning among parents were not explored. Despite repeated reminders and requests families did not visit and participate in the study. This study provides new insights into family planning among thalassemia minors, a previously unknown area in the region. The higher rates of cousin marriages in Kech potentially explain the elevated rates of thalassemia in the region.

## CONCLUSION

The religious beliefs, economic status of the family, area of residence, and total number of children play a significant role in family planning practices among the parents of children affected with BTM. The desire to have more children restrains the parents to practice family planning.

### Recommendations:

Thorough and prompt intervention is required in the community to raise the prevalence of family planning. Counseling and awareness about the prevention of thalassemia should be provided by authorities before the marriage and conception of a child.

### Authors’ Contribution:

**Uzair:** Conception and design of study, data collection and analysis, drafting the article. Taking the responsibility of integrity of study.

**MM:** Collection and interpretation of data, drafting the article.

**ZG:** Conceptualization of study, data collection, drafting article, and revising for intellectual content.

**JB:** Collection and interpretation of data, write-up.

**SKN:** Analysis and interpretation of data, critical revision of article, final approval for publication.

**NS:** Conception of study, final approval for publication.

All the authors are responsible and accountable for the accuracy and integrity of the work.
